# The *Candida albicans* Dse1 Protein Is Essential and Plays a Role in Cell Wall Rigidity, Biofilm Formation, and Virulence

**DOI:** 10.1155/2011/504280

**Published:** 2011-06-30

**Authors:** Jalil Y. Daher, Joseph Koussa, Samer Younes, Roy A. Khalaf

**Affiliations:** Natural Sciences Department, Lebanese American University, P.O. Box 36, Byblos, Lebanon

## Abstract

The fungal pathogen *Candida albicans* is one of the leading causative agents of death in immunocompromised individuals. It harbors an arsenal of cell wall anchored factors that are implicated in virulence such as filamentation inducing factors, adhesins, lipases, proteases, and superoxide dismutases. Dse1 is a cell wall protein involved in cell wall metabolism. The purpose of this study is to characterize the role Dse1 plays in virulence. Dse1 appears to be an essential gene as no homozygous null mutant was possible. The heterozygote mutant exhibited increased susceptibility to calcofluor white, a cell wall disrupting agent, with a subsequent reduction in cell wall chitin content, decreased oxidative stress tolerance, a 30% reduction in biofilm formation, and a delay in adhesion that was mirrored by a reduction in virulence in a mouse model of infection. Dse1 thus appears to be an important protein involved in cell wall integrity and rigidity.

## 1. Introduction

*Candida albicans *is the most common fungal pathogen in humans. This fungus exists as a commensal organism in healthy individuals by colonizing several niches of the human body [1]. These niches include skin and mucosal surfaces, oral cavity, vagina, and gastrointestinal tract. An altered balance between the host immunity and this opportunistic fungus, as in the case of immunocompromised patients, is one of the leading causes of candidiasis in humans. After entering the bloodstream, the yeast cells can infect all internal organs and may cause life-threatening septicemia [2]. In the USA, candidiasis is considered the third most common blood-borne infection and the fourth major cause of nosocomial infections. Candidiasis can develop as superficial candidiasis (skin and mucosa) which occurs in healthy individuals, or invasive candidiasis which is seen in cancer patients, AIDS patients, and immunocompromised individuals following transplantation [1]. The latter conditions have been shown to be associated with a high rate of mortality which can range between 35% and 60%. 

A remarkable characteristic of *C. albicans *is its ability to grow in two different forms: ellipsoidal buds (blastospores) or hyphae [3]. This asexual diploid fungus can form blastospores which are free floating cells that grow independently from each other or hyphae which are elongated cells attached to each other. The transition between the two forms can be stimulated by environmental factors such as body temperature (37°C), neutral to basic pH, or by some more critical filamentation inducing factors such as blood serum, hypoxia, physiological CO_2_ concentration, carbon and nitrogen starvation, and macrophage contact [3, 4]. This capability to switch between these two distinct morphologies is relevant to the pathogenicity of this organism since the filamentous form is necessary for invasive growth and the yeast form for clonal expansion.

Although candidiasis may arise from an immunologic dysfunction in the host organism, much credit should be given to the opportunistic pathogen itself, its ability to adapt to various niches, and its capability to express many crucial genes that are involved in infection. Such factors include adhesins [5, 6], lipases and phospholipases [7, 8], secreted aspartyl proteases [9], iron acquisition [10], and oxidative stress response [11]. Most of the above-mentioned factors are cell wall proteins attached to the the *β*-1,3 or 1,6 glucans, and face the exterior to interact with the environment. In addition to glucans the cell wall contains a stress tolerant innermost layer of chitin [12, 13]. As such *C. albicans* cell wall proteins have been the subject of intense characterization and are considered prime targets for novel antifungal drugs.

The purpose of the current study is to characterize Dse1, a 724 amino acid uncharacterized predicted cell wall protein whose transcription is decreased in a *C. albicans* mutant strain lacking the transcription factor Ace2. Ace2p is a master regulator of the cell wall metabolism in *C. albicans *and is involved in the regulation of transcription during G1 phase of mitotic cycle [14]. Characterization will be performed by generating a Dse1 mutant strain and comparing the phenotype of the null to the parental as far as filamentation, adhesion to human epithelial cells, biofilm formation, cell wall chitin content, virulence in a mouse model of disseminated candidiasis resistance to oxidative stress, and susceptibility to cell surface disrupting agents.

## 2. Materials and Methods

### 2.1. Strains, Cell Lines, and General Growth Conditions

The RM1000 *C. albicans* strain (*ura3Δ::*λ*imm434/ura3Δ::*λ*imm434his1::hisG/his1::hisG,* [15], which is auxotrophic for uridine and histidine, was used in this study. Cells were grown without selection on potato dextrose agar (PDA) medium (HiMedia, India). When selective growth was required, the yeast nitrogen base (YNB) synthetic medium was utilized (Fluka, Switzerland). Media was supplemented with 75 *μ*M uridine and histidine for all assays carried out to account for possible differences in levels of uridine expression. Images were generated utilizing an Olympus E330-ADU-1.2x stereomicroscope for colony morphology and an Olympus CX41 for cell imaging coupled with a Sony DSC-S40 digital camera. A Sony Cybershot Camera was used for colony spotting pictures. 

The human colon adenocarcinoma cell line HT-29 ATCC number HTB-38 was utilized in this study. Cells were grown on RPMI 1640 medium supplemented with 25 mM Hepes and L-glutamine with 10% FBS. 100 *μ*g/mL of streptomycin and 100 U/mL of penicillin were added, and cells were grown in a 5% CO_2_ incubator.

### 2.2. *Dse1* Knockout Generation

DNA cassettes containing the functional *URA3* or *HIS1* markers flanked by 100 bp *Dse1* sequences 5′ and 3′ were generated as previously described [16, 17] of homology. Homologous recombination and integration created a *DSE1/URA3::dse1* and a *DSE1/HIS1::dse1* heterozygous strain. 

### 2.3. Cassette Transformation

*C. albicans* strain RM1000 was transformed with the previously constructed *DSE1-URA3* and *DSE1-HIS1* cassettes by using a modified protocol of the Alkali Yeast Cation Transformation Kit (Q-Biogene, Germany) [18]. The RM 1000 parental strain was transformed with plasmid p*ABSK2* to restore uridine prototrophy as described previously [16, 17]. Successful transformants were selected on selective YNB medium supplemented with the appropriate amino acids. 

### 2.4. Filamentation Assay

Strains were grown on PDA for 14 days at 30°C and 37°C and then checked for filamentous and invasive growth on the agar medium. Furthermore, strains were grown in potato dextrose broth PDB and 100% liquid fetal bovine serum FBS (HiMedia) for 12 h at 30°C and 37°C and then observed under the microscope.

### 2.5. Antifungal Susceptibility Test

For caspofungin susceptibility determination, the *E*-test (AB Biodisk, Solna, Sweden) performed on RPMI agar supplemented with L-glutamine and 165 mM MOPS (BioWhittaker, Belgium) was performed according to the manufacturer's instructions, and the MIC was read after 48 h of incubation at 35°C. The *C. albicans* ATCC 90028 CLSI quality control reference strain was used in this study. The MICs of the control strains fell within the acceptable limits.

### 2.6. Susceptibility to Cell Wall Disrupting Agents

The wild type and *DSE1/dse1* mutant strains were grown in rich PDB media until exponential phase (OD_600_ = 0.6–0.8). Serial dilutions were performed (10^5^ to 10^2^ cells/mL), and a volume of 5 *μ*L was spotted on PDA plates containing either one of the following cell surface disrupting agents: sodium dodecyl sulfate at a concentration ranging from 0.02% to 0.05%, calcofluor white at a concentration ranging from 25 to 100 *μ*g/mL, and Congo red at a concentration ranging from 10 to 50 *μ*g/mL. Plates were incubated at 30°C and monitored for 4 days. Plates lacking the disrupting agents were used as controls [16].

### 2.7. Oxidative Stress Test

Strains were grown in rich potato dextrose broth media to exponential phase, then serial dilutions (10^5^ to 10^2^ cells/mL) were prepared and treated with hydrogen peroxide (10, 25 or 50 mM) for 1 hour followed by spotting 5 *μ*L onto PDA plates. Untreated cultures were also spotted as controls. Growth was monitored for 3 days [19].

### 2.8. Adhesion to Epithelial Cells

Adhesion to epithelial cells was assessed using the HT-29 human cell line and as previously described [20]. Briefly, strains were grown to exponential phase and subsequently diluted to 100 cells/mL that were added to six-well microtiter plates containing epithelial cells and incubated for 45 and 90 minutes at 37°C. After incubation, nonadherent cells were removed by washing the wells twice with PBS; the wells were then overlaid with molten PDA agar. After 24 hrs, adhesion to epithelial cells was evaluated by comparing the number of colonies present on the microtiter plates with colonies present on control PDA plates. Adhesion was expressed as a percentage of the original inoculum. 

### 2.9. Biofilm Assay

The assay was performed as previously described [21] with slight modification. Briefly, 500 *μ*L of a 10^7^ cells/mL culture was added onto the wells of a polystyrene microtiter plate pretreated with 0.05% fetal bovine serum for 24 h at 4°C. Plates were incubated in a shaker at 37°C and 75 rpm for 3 hrs after which nonadherent cells were washed away with PBS, 1 mL YNB was added to each well, and plates were reincubated for 48 h at 37°C. Plates were rinsed again with PBS, and 500 *μ*L of 99% methanol was added to each well to fix the newly formed biofilms. After 15 minutes, methanol was removed and plates were allowed to air dry. 500 *μ*L of a 0.2% crystal violet solution was added to each well, and the plate was incubated for 20 minutes. Finally, unbound crystal violet was washed away with distilled water, and bound crystal violet was released with 750 *μ*L of a 33% acetic acid solution. Absorbance of the released crystal violet solution was read at 590 nm. A negative control with no cells was also performed.

### 2.10. Disseminated Candidiasis Assay

Eighteen 20–30 grams BALB/c mice were injected through the lateral tail vein with 5 × 10^6^ *C. albicans* cells. Mice were monitored for survival over a period of three weeks. Food and water were given *ad libitum* [4].

### 2.11. Chitin Quantification

Cell wall chitin content was measured according to a modified protocol described previously [13, 22]. Briefly, 6N HCl was used to hydrolyze 50 mg wet weight purified cell walls at 100°C overnight. After centrifugation, the pellet was reconstituted in 1 mL of distilled water. A 0.1 mL aliquot of this sample was added to 0.1 mL of solution A (1.5 N Na_2_CO_3_ in 4% acetylacetone). The mixture was incubated at 100°C for 20 minutes, and after cooling to room temperature, 0.7 mL of 96% ethanol was added to the mixture followed by addition of a 0.1 mL of solution B (1.6 g of *p*-dimethyl-aminobenzaldehyde in 30 mL of concentrated HCl and 30 mL of 96% ethanol). The mixture was incubated for 1 hour at room temperature, and absorbance of the samples was measured spectrophotometrically at 520 nm. The results were plotted against a standard curve generated by the use of known glucosamine standards taken through the same procedure as our samples. Chitin amount was expressed as a percentage of the wild-type strain.

### 2.12. Homology Assessment

The *C. albicans* database (http://www.candidagenome.org/) was used to retrieve the sequence of the Dse1 gene. Performing a BLAST search of the Dse1 ORF onto the *Saccharomyces cerevisiae,* database (http://www.yeastgenome.org/) revealed the closest *S. cerevisiae* orthologue

### 2.13. Statistical Analysis

All assays were done in triplicates. The multiple comparison LSD (least square differences) test was used to analyze the results of the biofilm assay. For the virulence assay, survival analysis was based on the Kaplan Meier method and the log Rank test. The student *t*-test was used for the adhesion assay, and for chitin content analysis of variance (ANOVA) was carried out with multiple comparison technique to control for alpha inflation. *P* value less than 5% was considered significant.

## 3. Results

### 3.1. Deletion Generation

The RM1000 parental strain was transformed with the 1513 bp *URA3* cassette or a 1420 bp *HIS1* cassette. One *URA3 *cassette underwent homologous recombination at one of the two native Dse1 allele sites resulting in the formation of *a DSE1/dse1::URA3 *heterozygote strain. Similarly, one *HIS1* cassette was integrated at the Dse1 locus generating a *DSE1/dse1::HIS1* strain. To screen for successful transformation, the cells were selected by growth on YNB medium lacking uridine, and/or histidine. For verification of correct integration a primer that hybridized outside the Dse1 integration cassette locus and a reverse primer that hybridized inside the *URA3* or *HIS1* gene were used for amplification. To confirm the absence or presence of any Dse1 alleles, a PCR reaction with Dse1 internal primers was carried out (data not shown).

### 3.2. Filamentation Assay

**Figure 1:**
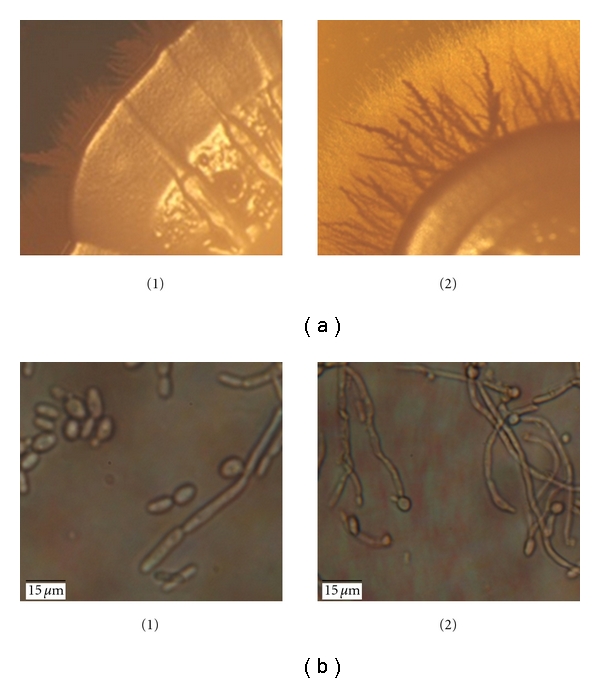
Colony morphology on solid and liquid media. (a) Colony morphology of both wild-type (1) and heterozygote mutant (2) strains grown on PDA at 30°C clearly indicates an increase in filamentation and invasivness of the mutant compared to the wild type. (30x magnification). (b) Cell morphology in PDB media after 24 h incubation at 37°C (1000x magnification).

The wild-type parental strain RM1000 harboring the p*ABSK2* plasmid and the *DSE1/dse1::URA3* heterozygote mutant strains were grown on PDA plates for 14 days at 30°C. The mutants showed a marked difference in filamentation and invasiveness compared to the wild-type strain as the filaments were longer and penetrated deeper into the agar. The mutant was thus hyperfilamentous (Figure 1(a)). This difference was also mirrored in liquid PDB media after overnight growth at 37°C (Figure 1(b)). No difference on FBS was observed as both strains filamented equally well (data not shown).

### 3.3. Antifungal Susceptibility Test

The *E*-test method was used to determine the sensitivity of the mutant heterozygote strain to the antifungal agent caspofungin. After 48 h of incubation, the mutant strain showed no significant discrepancy compared to the wild type (Table 1).

### 3.4. Adhesion Assay

**Figure 2:**
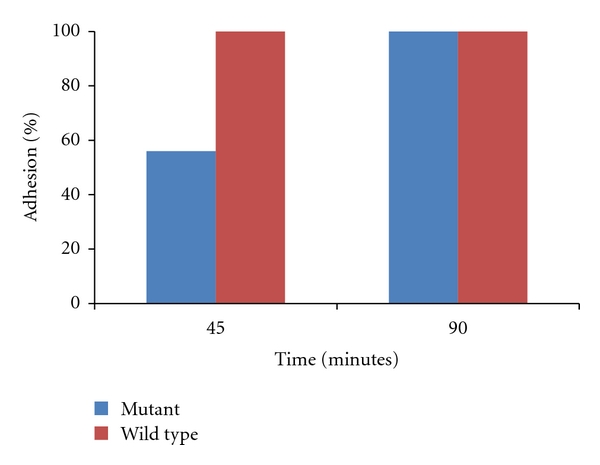
Adhesion to human epithelial cells. Strains were incubated with cell line HT-29 for 45 and 90 minutes. After washing away, any nonadherentcells wells were overlaid with molten PDA. Adhesion to epithelial cells was assessed by counting the number of colonies present on the microtiters plates after 24 h and comparing to control plates. Adhesion was expressed as a percentage of the original inoculums. As can be seen, the mutant exhibited a significant delay in adhesion after 45 min (*P* < .001).

Strains were assayed for adhesion to the human epithelial cell line HT-29 for 45 and 90 minutes. After 45 minutes, there was a clear and significant delay in adhesion of the mutant heterozygote strain as compared to the wild-type strain (*P* value less than  .001). However, after 90-minute incubation, the wild type and mutant exhibited similar adhesion capabilities. Thus, the mutant exhibits an adhesion delay phenotype rather than an adhesion defect (Figure 2). 

### 3.5. Biofilm Assay

**Figure 3:**
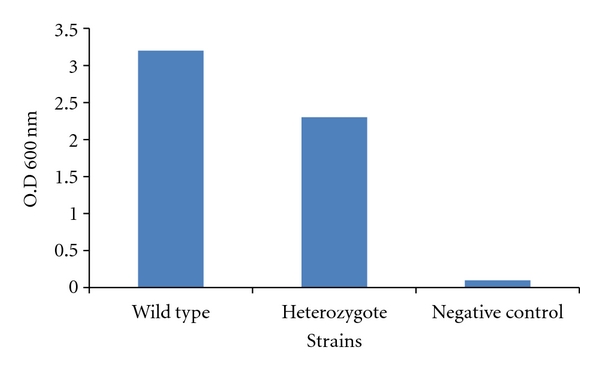
Biofilm formation on polystyrene surfaces. Strains were plated on polystyrene microtiter plates and incubated for 24 h followed by rinsing and methanol addition to allow the fixation of biofilm-forming cells. Afterward, crystal violet was added to stain the newly formed biofilm cells. Unbound crystal violet was washed away and bound crystal violet was released by adding acetic acid to the wells. OD at 600 nm of the released crystal violet was read, and it proportionally corresponded to the amount of biofilm forming cells present in the wells. The negative control lacked any *C. albicans* cells but was treated similarly. Note the significant defect in biofilm formation in the mutant strain (*P* = .002).

Strains were assayed for biofilm formation capabilities on plastic polystyrene surfaces. The heterozygote mutant was significantly defective in biofilm formation by 30% as compared to the wild type (*P* = .002) (Figure 3).

### 3.6. Mouse Model of Disseminated Candidiasis

**Figure 4:**
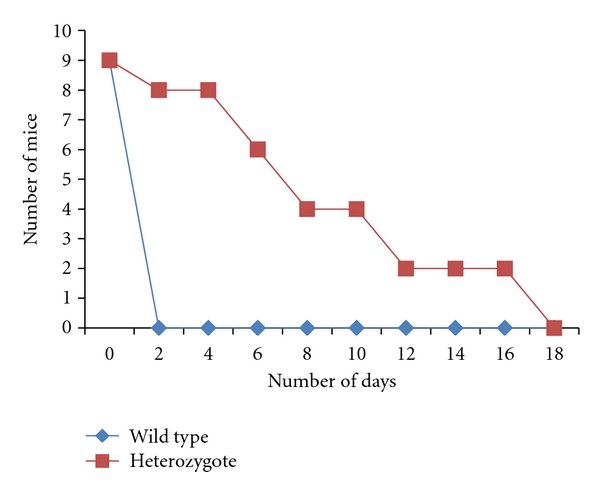
Disseminated model of candidiasis. BALB/c mice were injected in their tail vein with 5 × 10^6^ *C. albicans* cells, and survival was monitored daily. Survival analysis was based on the Kaplan Meier method and the log Rank test. As can be seen, the mutant strain is significantly defective in virulence when compared to the wild type (*P* = .001).

Nine mice/strains were injected via the tail vein with 5 × 10^6^ *C. albicans* cells. Mice were monitored for a period of 3 weeks for survival. Mice injected with the mutant cells survived for a significantly longer period when compared to mice injected with the wild-type cells implying a decrease in virulence for the mutant strain (*P* = .001). The median survival was 1.75 days for the wild type and 8 days for the heterozygote (Figure 4).

### 3.7. Chitin Content

**Figure 5:**
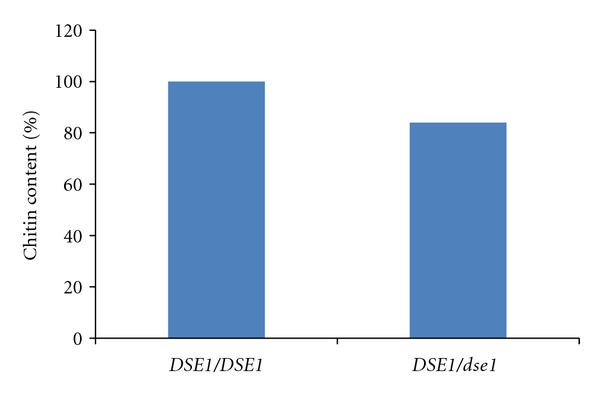
Chitin content. Cell wall chitin content was measured as absorbance at OD600 of N-acetylglucosamine monomers released after HCl hydrolysis of 50 mg purified cell walls. Mutant chitin content was 84% that of the wild type (*P* = .02).

Cell wall chitin was quantified spectrophotometrically by the release of N-acetylglucosamine monomers after acid hydrolysis of purified cell walls. Note the statistically significant (*P* = .02) decrease in chitin content in the mutant compared to the wild type (Figure 5).

### 3.8. Oxidative Stress Assay

**Figure 6:**
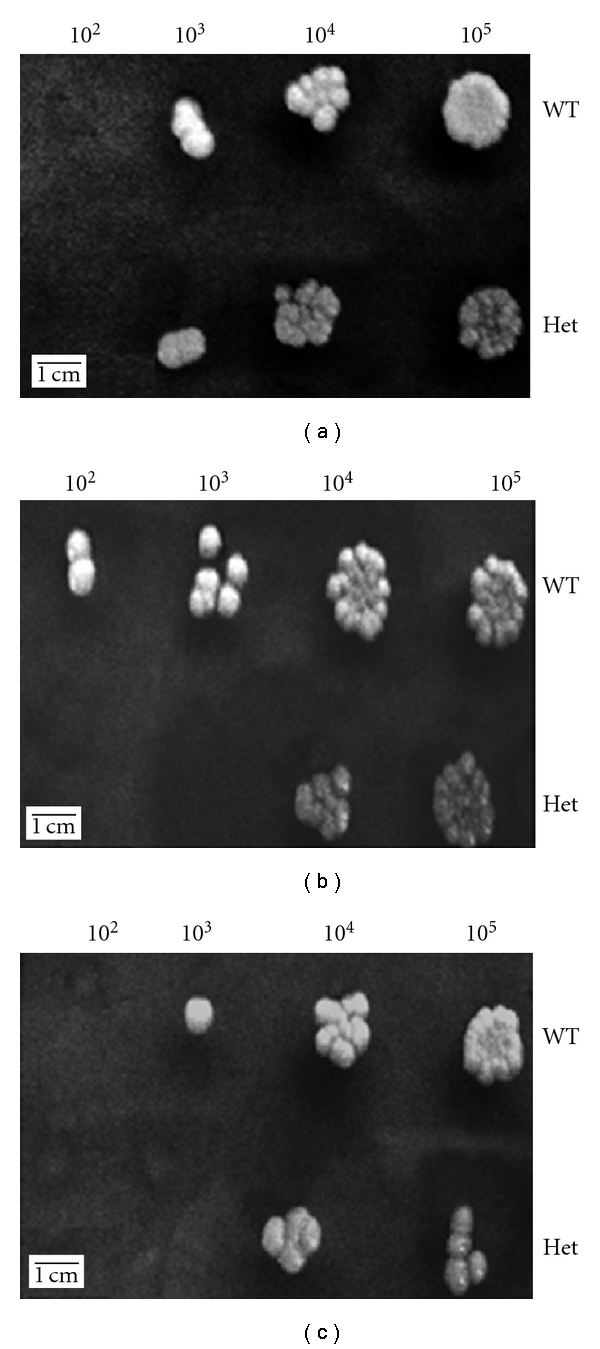
Oxidative stress assay. (a) Control plate; (b) 10 mM concentration of H_2_O_2_; (c) 25 mM concentration of H_2_O_2_. Strains were grown in PDB, then diluted (10^5^ to 10^2^ cells/mL), and incubated with different doses of hydrogen peroxide for one hour. 5 *μ*L of the treated culture was spotted on PDA plates. Untreated cultures were also spotted as controls. Note the increased susceptibility of the mutant to hydrogen peroxide.

The wild-type strain and the *DSE1/dse1::URA3* heterozygote mutant strains were subjected to various doses of hydrogen peroxide and spotted on PDA plates. The mutant strain showed a decrease in resistance to oxidative stress when compared to the wild type (Figure 6).

### 3.9. Cell Surface Disturbing Agents

**Figure 7:**
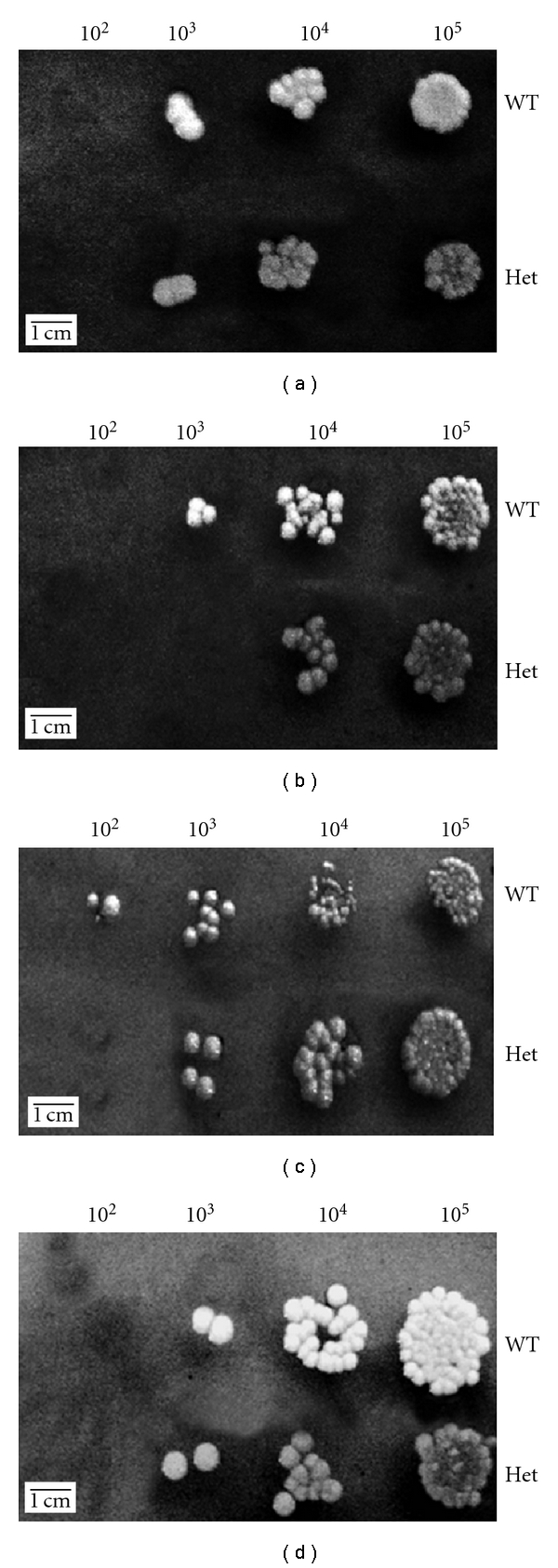
Cell surface disrupting agents. (a) Control plate; (b) 12.5 *μ*g/mL calcofluor white; (c) 0.0225% SDS; (d) 50 *μ*g/mL Congo red. Strains were grown in PDB, then diluted (10^5^ to 10^2^ cells/mL), and spotted on PDA plates containing different concentrations of cell surface disrupting agents. A slight increase in susceptibility to SDS and calcofluor white was observed.

The wild-type and the mutant strains were grown in the presence of different concentrations of various cell surface disrupting agents. A control plate contained the same dilutions of both strains but with no disrupting agents added was also performed. The mutant strain was found to be slightly more susceptible to calcofluor white and SDS when compared to the wild type with no difference in susceptibility to Congo red (Figure 7). 

## 4. Discussion

In this study, we characterized Dse1, a predicted *C. albicans* cell wall protein. The reason behind our choice of this specific cell wall protein was that even though Dse1 has not yet been characterized, Ace2, its transcriptional regulator, and the *Saccharomyces cerevisiae* Dse1 orthologue have both been. In *Candida,* an *ace2/ace2* knockout strain exhibits many interesting phenotypes including an increase in filamentation and agar invasion, a cell separation defect, a reduction in adherence, a sharp decrease in biofilm formation [14], and avirulence in a mouse model of infection [23]. The deletion of the *ACE2 *gene reduces the expression of two cell wall genes Dse1 and *SCW11 *in both yeast and hyphal form. The deletion has also an effect on the *CHT3* gene encoding a chitinase enzyme which is involved in chitin production in the cell [14]. In *Candida*, the Dse1 gene also shows a pattern of periodic expression with a peak expression at the M/G1 phase [24]. The *CaDSE1* orthologue in *S. cerevisiae *is *ScDSE1 *which is specifically a daughter cell protein, and it is involved in pathways controlling cell wall metabolism. Additionally, a mutant *S. cerevisiae *strain lacking the Dse1 gene is defective in cell separation [25, 26]. An alignment of CaDse1p and ScDse1p revealed a 53% sequence homology between the two proteins. 

Since *C. albicans* is a diploid organism, characterization of Dse1 function required knocking out both alleles. An interesting outcome of our study was our inability to generate a *dse1 *homozygous null strain. Twenty-six colonies from 3 independent transformations of the *DSE1/dse1::HIS1* heterozygote with the *URA3* cassette were obtained. In all the twenty-six screened colonies, the presence of a Dse1 internal PCR product indicated a failure in the generation of a homozygous null strain. On the other hand, 3 out of 5 colonies of the parental RM1000 strain transformed with the *HIS1* cassette had the cassette integrated at the correct site and were thus heterozygous. This observation implied the possibility that the problem may have been in the *URA3* cassette itself rendering it unable to successfully integrate at the correct Dse1 locus and thus preventing the generation of a *Dse1* null strain. To address this problem, we attempted to recreate the Dse1 heterozygote strain but by transforming the parental strain with the *URA3* cassette first. Such a heterozygote was successfully generated (5 successful integrations out of 5 colonies screened). The fact that each cassette was independently transformed successfully but the combination could not strongly imply that the Dse1 gene is essential. This conclusion is supported by failure of generation of a Dse1 null strain from another lab group utilizing a different technique, the *UAU1* cassette transformation (http://www.candidagenome.org/ unpublished data). As such, the presence of a Dse1 allele in all transformants with two *Dse1* alleles knocked out is a further proof of the essentiality of the protein. Such a result is interesting and quite unpredictable since an *ace2* null strain does exist and Ace2 is the only known activator of Dse1, suggesting other uncharacterized pathways of Dse1 activation.

Since a heterozygote strain with only one copy of a particular gene usually produces less functional protein than a wild-type strain with two functional alleles, a haploinsufficiency phenotype is common in *C. albicans*. This is true of many cell wall proteins. For instance, a *C. albicans* mutant strain, heterozygote for the *DDR48* gene, has been shown to be haploid insufficient as it was defective in filamentation, hypersensitive to oxidative stress, and reduced levels of drug resistance [4]. Similarly, an *SOD5* heterozygote strain was shown to be haploinsufficient and was more susceptible to oxidative stress and less virulent in a mouse model of infection [27]. In our study, we determined the phenotypic difference between the ura3^+^ heterozygote mutant strain and the wild-type parental strain, and we found that our mutant strain exhibited a significant degree of haploinsufficiency. 

It was previously documented that the outcome of cell wall mutations in *C. albicans* is a weakened cell wall, and in most cases of cell wall protein deletions, the resulting cell tends to be more susceptible to antifungal agents, drugs, and oxidative stress [4]. Such data agrees with the results of our study concerning a more weakened cell surface. For instance, discrepancies were observed between the mutant and the wild type upon incubation with calcofluor white, a drug that targets exclusively the *C. albicans* cell wall, with the mutant exhibiting an increase in sensitivity. Interestingly, however, there was no difference between our mutant and the wild type concerning sensitivity to Congo red and caspofungin even though these two drugs also target the cell wall. A possible explanation of this phenomenon is that the mechanism of action of calcofluor white is somewhat different from that of Congo red and caspofungin. Calcofluor white affects chitin assembly within the cell wall, whereas Congo red and caspofungin alter glucan synthesis and assembly [28–30]. This hypothesis is further strengthened by our finding that chitin deposition is also slightly but statistically significantly decreased in the mutant resulting in a less rigid cell wall. The mutant was also found to be more sensitive to SDS and to oxidative stress when compared to the wild type. SDS is known to disrupt and solubilize the plasma membrane and is thus not a cell wall-specific compound [31]. However by weakening the cell wall, permeability to SDS might have increased rendering the cell more susceptible. As far as hydrogen peroxide is concerned, one possible explanation is that by decreasing the amount of Dse1 on the cell surface, the architecture of the cell wall proteome might have been changed possibly preventing correct positioning and anchoring of cell wall localized superoxide dismutases or other proteins that are directly or indirectly responsible for countering oxidative stress damage [32].

The above-mentioned rational might also explain the defect observed in biofilm formation, as changes to the cell wall proteome brought on by the *Dse1* deletion and subsequent weakening of the cell wall might have hindered the ability of the strain to aggregate and form proper biofilm. Surprisingly, the adhesion data does not go hand in hand with our biofilm data. A defect in biofilm can be in most cases explained as due to a defect in adhesion, the first and necessary step for biofilm formation to proceed [33]. However, the adhesion data showed that the mutant strain is not defective in adhesion; it rather displays a delay in adhesion. Whether this delay affects biofilm formation is debatable. The defect in biofilm formation might be due to other factors besides adhesion to plastic factors that are needed in advanced stages of biofilm formation that might involve protein aggregation or cell-to-cell recognition and adhesion.

Furthermore, after testing the degree of virulence of our mutant in a mouse model, it was observed that the mutant was decreased in virulence when compared to the wild type. While the wild-type strain was able to kill all mice within two days after injection, the mutant strain took more than two weeks to kill the mice. Although the mutant strain was eventually able to kill all mice, the delay is statistically significant and implies attenuated virulence. Bearing in mind that virulence highly correlates with adhesion and vice versa [34], our results show that the delay in adhesion and defect in biofilm formation was mirrored by a decrease in virulence. One possible explanation may be that the delay in adhesion allows for a crucial amount of time for the immune system to get rid of the fungus [20]. 

Our results show similarity with those of an *ace2* null. However, the phenotype exhibited by the *ace2/ace2* mutant is more severe and can be explained by the fact that Ace2 controls not just Dse1 but many proteins implicated in cell wall metabolism. In addition, the *ace2* null exhibited an increased invasion on solid media and filamentation. Such a phenotype was also mirrored in our *Dse1* null strain as our strain was overfilamentous on both solid and liquid media compared to the parental strain with an increase in invasion of solid agar after a fourteen-day incubation period that allows for glucose depletion, a positive trigger of filamentation [17]. No explanation was given for such a phenotype. Whether the cell upregulates filamentation genes to compensate for the *Dse1* deletion remains to be determined. 

Additionally, a Dse1 orthologue named *ScDSE1* is also found in *S. cerevisiae *and encodes a protein that has been shown to participate in pathways regulating cell wall metabolism. A *Dse1* deletion in *S. cerevisiae* renders the mutant cells more sensitive to cell wall targeting drugs [25, 26], a phenotype similar to what we observed with our mutant. However, again, and as is the case with the *ace2* knockout strain, the *Scdse1* null phenotype was more severe than the one observed for the mutant in our study. Again it is important to keep in mind here that both the *ace2 *and* Scdse1* mutant strains were homozygous nulls. We were unable to generate such a null strain, and as such we are comparing a null strain with a heterozygote strain that is still producing a functional protein. Bearing this in mind, it is not surprising that our phenotype is more subtle.

## 5. Conclusion

Dse1 appears to be an essential *C. albicans* cell wall protein and as such a potential candidate for novel antifungal drugs. Knocking out one copy of the gene renders the strain haploinsufficient. This haploinsufficiency manifests itself as a decrease in resistance to caspofungin, a cell wall disrupting agent, through weakening the cell wall by diminishing the amount of chitin deposited, a decrease in resistance to oxidative stress, delayed adhesion, decreased ability to form biofilm, and a subsequent reduction in virulence. Future studies should be carried out to answer some core issues related to this study. In order to explain the hyperfilamentous phenotype, microarray analysis of the mutant would be very useful to assess which filamentation pathways may have been modified to compensate for the deletion. Moreover, MALDI-TOF mass spectrometric analysis of the cell surface proteome would be essential in providing us with some insights as to what changes have occurred in the cell surface architecture leading to the observable phenotype. Such experiments would be valuable in completing our study resulting in further characterization of Dse1 and the exact role it plays in *C. albicans.*

## Figures and Tables

**Table 1:** Caspofungin susceptibility. The MIC of caspofungin was measured by the *E*-test method. No significant difference between the wild-type parental and the mutant was observed.

Strains	MIC (*μ*g/mL)
Wild type	0.125
Heterozygote	0.094

## References

[1] Larriha G, Rubio Coque JJ, Ciudad A, Andaluz E (2000). *Candida albicans* molecular biology reaches its maturity. *International Microbiology*.

[2] Karkowska-Kuleta J, Rapala-Kozik M, Kozik A (2009). Fungi pathogenic to humans: molecular bases of virulence of Candida *albicans*, *Cryptococcus neoformans* and *Aspergillus fumigatus*. *Acta Biochimica Polonica*.

[3] Molero G, Díez-Orejas R, Navarro-Garcîa F (1998). *Candida albicans*: genetics, dimorphism and pathogenicity. *International Microbiology*.

[4] Dib L, Hayek P, Beyrouthy B, Khalaf RA (2008). The *C. albicans* Ddr48 protein is essential for filamentation, stress response & confers partial antifungal drug resistance. *Medical Science Monitor*.

[5] Daniels KJ, Lockhart SR, Staab JF, Sundstrom P, Soll DR (2003). The adhesin Hwp1 and the first daughter cell localize to the a/a portion of the conjugation bridge during *Candida albicans* mating. *Molecular Biology of the Cell*.

[6] Li F, Svarowsky MJ, Karlsson AJ (2007). Eap1p, an adhesion that mediates *Candida albicans* biofilm formation in vitro and in vivo. *Eukaryotic Cell*.

[7] Yang YL (2003). Virulence factors of *Candida* species. *Journal of Microbiology, Immunology and Infection*.

[8] Schaller M, Borelli C, Korting HC, Hube B (2005). Hydrolytic enzymes as virulence factors in *Candida albicans*. *Mycoses*.

[9] Naglik JR, Challacombe SJ, Hube B (2003). *Candida albicans* secreted aspartyl proteinases in virulence and pathogenesis. *Microbiology and Molecular Biology Reviews*.

[10] Almeida RS, Brunke S, Albrecht A (2008). The hyphal-associated adhesin and invasin Als3 of *Candida albicans* mediates iron acquisition from host ferritin. *PLoS Pathogens*.

[11] Brown AJP, Odds FC, Gow NA (2007). Infection-related gene expression in *Candida albicans*. *Current Opinion in Microbiology*.

[12] Chaffin WL, Lopez-Ribot JL, Casanova M, Gozalbo D, Martinez J (1998). Cell wall and secreted proteins of *Candida albicans*: identification, function, and expression. *American Society of Microbiology*.

[13] Kapteyn JC, Hoyer LL, Hecht JE (2000). The cell wall architecture of *Candida albicans* wild-type cells and cell wall-defective mutants. *Molecular Microbiology*.

[14] Kelly MT, MacCallum DM, Clancy SD, Odds FC, Brown AJ, Butler G (2004). The *Candida albicans CaACE2* gene affects morphogenesis, adherence and virulence. *Molecular Microbiology*.

[15] Negredo A, Monteoliva L, Gil C, Pla J, Nombela C (1997). Cloning, analysis and one-step disruption of the *ARG5,6* gene of *Candida albicans*. *Microbiology*.

[16] Hashash R, Younes S, Bahnan W, El Koussa J, Maalouf K, Khalaf RA Characterization of Pga1, a *Candida albicans* cell wall protein necessary for proper adhesion and biofilm formation.

[17] Hayek P, Yazbek P, Beyrouthy B, Khalaf RA (2009). Characterization of *HWP2*, a *Candida albicans* putative GPI-anchored cell wall protein necessary for invasive growth. *Microbiological Research*.

[18] Khalaf RA, Zitomer RS (2001). The DNA binding protein Rfg1 is a repressor of filamentation in *Candida albicans*. *Genetics Society of America*.

[19] Pedreño Y, González-Párraga P, Martínez-Esparza M, Sentandreu R, Valentín E, Argüelles J (2007). Disruption of the *Candida albicans ATC1* gene encoding a cell-linked acid trehalase decreases hypha formation and infectivity without affecting resistance to oxidative stress. *Microbiology*.

[20] Tsuchimori N, Sharkey LL, Fonzi WA, French SW, Edwards JE, Filler SG (2000). Reduced virulence of *HWP1*-deficient mutants of *Candida albicans* and their interactions with host cells. *Infection and Immunity*.

[21] Peeters E, Nelis HJ, Coenye T (2008). Comparison of multiple methods for quantification of microbial biofilms grown in microtiter plates. *Journal of Microbiological Methods*.

[22] Munro CA, Whitton RK, Hughes HB, Rella M, Selvaggini S, Gow NA (2003). *CHS8*-a fourth chitin synthase gene of *Candida albicans* contributes to in vitro chitin synthase activity, but is dispensable for growth. *Fungal Genetics and Biology*.

[23] Noble SM, French S, Kohn LA, Chen V, Johnson AD (2010). Systematic screens of a *Candida albicans* homozygous deletion library decouple morphogenetic switching and pathogenicity. *Nature Genetics*.

[24] Côte P, Hogues H, Whiteway M (2009). Transcriptional analysis of the *Candida albicans* cell cycle. *Molecular Biology of the Cell*.

[25] Colman-Lerner A, Chin TE, Brent R (2001). Yeast Cbk1 and Mob2 activate daughter-specific genetic programs to induce asymmetric cell fates. *Cell*.

[26] Doolin MT, Johnson AL, Johnston LH, Butler G (2001). Overlapping and distinct roles of the duplicated yeast transcription factors Ace2p and Swi5p. *Molecular Microbiology*.

[27] Martchenko M, Alarco A, Harcus D, Whiteway M (2004). Superoxide dismutases in *Candida albicans*: transcriptional regulation and functional characterization of the hyphal-induced *SOD5* gene. *Molecular Biology of the Cell*.

[28] Elorza MV, Rico H, Sentandreu R (1983). Calcofluor white alters the assembly of chitin fibrils in *Saccharomyces cerevisiae* and *Candida albicans* cells. *Journal of General Microbiology*.

[29] Kopecka M, Gabriel M (1992). The influence of Congo red on the cell wall and (1,3)-*β*-d-glucan microfibril biogenesis in *Saccharomyces cerevisiae*. *Archives of Microbiology*.

[30] Lesage G, Sdicu AM, Ménard P, Shapiro J, Hussein S, Bussey H (2004). Analysis of *β*-1,3-glucan assembly in *Saccharomyces cerevisiae* using a synthetic interaction network and altered sensitivity to caspofungin. *Genetics*.

[31] Plaine A, Walker L, Da Costa G (2008). Functional analysis of *Candida albicans* GPI-anchored proteins: roles in cell wall integrity and caspofungin sensitivity. *Fungal Genetics and Biology*.

[32] Hwang C, Rhie G, Oh J, Huh W, Yim H, Kang S (2002). Copper- and zinc-containing superoxide dismutase (Cu/ZnSOD) is required for the protection of *Candida albicans* against oxidative stresses and the expression of its full virulence. *Microbiology*.

[33] Chandra J, Kuhn DM, Mukherjee PK, Hoyer LL, McCormick T, Ghannoum MA (2001). Biofilm formation by the fungal pathogen *Candida albicans*: development, architecture, and drug resistance. *Journal of Bacteriology*.

[34] Gale CA, Bendel CM, McClellan M (1998). Linkage of adhesion, filamentous growth, and virulence in *Candida albicans* to a single gene. *Science*.

